# The effect of computer guided total hip replacement on risk of revision, Oxford Hip Score, and health related quality of life: an analysis of National Joint Registry data

**DOI:** 10.1007/s00590-025-04622-9

**Published:** 2025-12-24

**Authors:** Muhamed M. Farhan-Alanie, Daniel Gallacher, Peter Craig, James Griffin, Jakub Kozdryk, James Mason, Peter D. H. Wall, J. Mark Wilkinson, Andrew Metcalfe, Pedro Foguet

**Affiliations:** 1 University of Warwick, Coventry, UK; 2 University of Birmingham, Birmingham, UK; 3 Worcestershire Acute Hospitals NHS Trust, Worcester, UK; 4 University Hospitals Coventry and Warwickshire NHS Trust, Coventry, UK; 5 The Royal Orthopaedic Hospital NHS Foundation Trust, Birmingham, UK; 6 University of Warwick, Coventry, UK; 7 School of Medicine and Population Health, The University of Sheffield, Sheffield, UK; 8 University Hospitals Coventry and Warwickshire NHS Trust, Coventry, UK

**Keywords:** Osteoarthritis, Hip replacement, Arthroplasty, Computer assisted surgery, Surgical navigation, Revision, PROMs

## Abstract

**Background:**

Total hip replacement (THR) can be performed conventionally or with the assistance of computer guidance systems. We aimed to compare the risk of revision for all-causes and dislocation, and differences in Oxford Hip Score (OHS) and health-related quality of life (EQ-5D-3L) following primary THR performed conventionally versus with the assistance of computer guidance systems.

**Methods:**

We performed an observational study using National Joint Registry data. Adult patients who underwent primary THR for osteoarthritis between 2003 and 2020 were included. The co-primary analyses were revision for all-causes and dislocation. Secondary analyses were differences in OHS and EQ-5D-3L. Weights based on propensity scores were generated. Cox proportional hazards and generalised linear models were used to assess outcomes of revision, OHS, and EQ-5D-3L. Effective sample sizes (ESS) were computed.

**Results:**

Risk of revision for all-causes comparing computer guided and conventional THR were similar (HR 0.947, 95% CI 0.698–1.283, *p* = 0.726, ESS 7235). However, sensitivity analysis restricting to the five most commonly used combination of prosthesis brands demonstrated reduced revision risk in favour of computer guidance (HR 0.446, 95% CI 0.231–0.858, *p* = 0.016, ESS 3993). There was no difference in revision for dislocation between groups (HR 0.929, 95% CI 0.512–1.688, *p* = 0.810, ESS 7235). Compared to conventional THR, the use of computer guidance increased OHS by 0.931 (95% CI 0.308–1.554, *p* = 0.003, ESS 2112) however there were no differences in EQ-5D-3L (0.007, 95% CI −0.008–0.023, *p* = 0.356, ESS 2929). Incidence of intra-operative complications was significantly fewer during computer guided THR (0.51% versus 0.96%, *p* = 0.006).

**Conclusions:**

There were no differences in revision for all-causes and dislocation between computer guided and conventional THR. However, sensitivity analysis considering only the five most commonly used prosthesis brands demonstrated a reduced risk of revision for all-causes favouring computer guided THR. Furthermore, computer guidance was associated with a significant but minimal improvement in OHS and a lower risk of intra-operative complications, without differences in EQ-5D-3L. Although these findings suggest potential for computer guided THR to improve implant survivorship and reduce intra-operative complications, they require cautious interpretation given the limitations inherent to observational study designs and registry based analyses.

**Supplementary Information:**

The online version contains supplementary material available at 10.1007/s00590-025-04622-9.

## Introduction

Total hip replacement (THR) is established as a very successful procedure for improving function and relieving pain in patients suffering from osteoarthritis [1, 2]. However, there remains scope for improvement in patient satisfaction and prosthesis longevity. Approximately 10% of patients report not being satisfied with the outcome of their procedure [1, 3] and THR survival is estimated to be 92.19% (95% confidence interval 92.02 to 92.35) at 18 years post-operatively [4]. Long term survivorship of prostheses is particularly relevant due to an increasing life expectancy and aging population, and their consequential effects on the incidence of costly revision arthroplasty procedures. Furthermore, patient reported outcomes following revision procedures are relatively inferior to those of primary THR surgery [5–8]. In an attempt to help address these issues, computer guidance systems have been introduced to allow the surgeon to assess component positioning with reference to anatomical landmarks in real time. Compared to conventional surgery that relies on surgeon’s experiential judgment and anatomic landmarks, relatively improved precision of component positioning and more consistent restoration of leg length and appropriate offset can be achieved with the use of computer guidance systems [9–12]. This may help to improve hip biomechanics and mitigate the risks of complications such as dislocation and accelerated implant wear due to component malpositioning. This should theoretically lead to improved functional outcomes and a reduced risk of future revision [13–18].

However, uncertainty remains as to whether the hypothesised benefits of computer guided surgery have materialised clinically. Previous registry-based studies on this topic that have examined outcomes revision, Oxford Hip Score, and health-related quality of life did not account for confounding by indication through statistical techniques. Furthermore, these studies were limited by small sample sizes, short follow-up periods, minimal adjustment for confounding variables, and focussed on a single implant and specific computer navigation system [19–21]. To address the limitations of previous research, the present study employed propensity score-based risk adjustment methods to minimise confounding and aimed to compare the risk of revision for all-causes and dislocation, and differences in Oxford Hip Score (OHS) and health-related quality of life (EQ-5D-3L) following primary THR performed using computer guidance versus conventional technique.

## Methods

### Study design and setting

We performed an observational study using data from the National Joint Registry (NJR) for procedures performed in England [4], National Health Service (NHS) England Patient Reported Outcome Measures (PROMs) programme [22], and Office for National Statistics (ONS) death data [23]. The NJR is a prospective register of primary and revision arthroplasty procedures. Data is contemporaneously submitted by the surgeon using a standardised form and has been mandatory in both the independent and public sectors since 2003 and 2011 respectively. Since April 2009, NHS funded patients undergoing elective primary THR in England are asked to complete the Oxford Hip Score (OHS) and Health Related Quality of Life (EQ-5D-3L) patient-reported outcome questionnaires preoperatively and six months post-operatively [4]. The OHS measures hip function and pain, while the EQ-5D-3L assesses quality of life across five dimensions (mobility, self-care, usual activities, pain/discomfort and anxiety/depression) [24, 25]. As this PROMs programme is administered by NHS England, the PROMs analysis was limited to NHS-funded procedures performed in England.

### Participants

All adult patients (≥ 18 years) who underwent primary THR for osteoarthritis only were eligible for inclusion. We excluded THR procedures with a metal-on-metal bearing combination due to their known high failure rates [26]. Based on a suggested reporting framework for PROMs, we analysed pre-operative and post-operative questionnaires if completed by patients within 18 weeks prior to surgery and within six to twelve months after surgery, respectively [4]. Patients who had died or underwent a revision procedure within twelve months of their initial procedure were excluded from the PROMs analyses, as inability to complete questionnaires or experiencing a revision procedure may confound their scores [4, 8]. The base dataset provided for analysis comprised 1,196,317 procedures performed between 1st April 2003 to 31st December 2020.

### Description of treatment/surgery

The exposures were conventional technique versus computer guided THR. Procedures were categorised under their respective groups based on the surgeon’s selection of these available options when completing the Minimum Data Set form after each procedure.

### Descriptive data

**Fig. 1:**
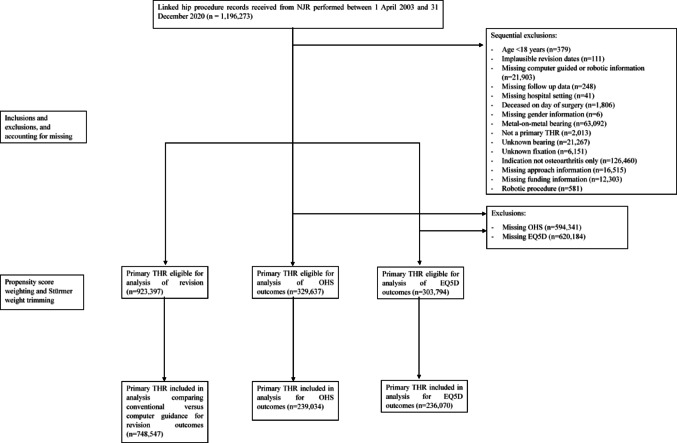
Flow diagram illustrating the process of inclusion and exclusion of procedure records

The flow of patient data through to data analysis is shown in Fig. 1. Following exclusions, there were no remaining procedures recorded using Minimum Data Set Version 1 which did not capture information on BMI and whether computer guidance was used.

The characteristics of the patient groups pre-weighing are shown in Table 1. Most procedures within the data set were performed using conventional technique (99.57% versus 0.43%). Median follow up time was relatively shorter for computer guided THR (4.73 versus 6.07 years). Mean age and ASA classification of patients were similar between groups however the computer guided group included a relatively higher proportion of male patients (43.8% versus 39.4%). Surgeons performed posterior approach more commonly when using computer guided systems compared to conventional technique (78% versus 63.6%). A greater proportion of conventional THR procedures involved use of a Metal-on-Polyethylene bearing (62% versus 52.7%). The most common implant fixation method was cementless in computer guided THR (59.4%) while most conventional THR procedures were performed using either cemented (35.6%) or cementless (39.3%) fixation. A relatively greater proportion of privately funded procedures involved the use of computer guidance (40% versus 14.7%). The proportion of records with missing BMI data were similar between groups (30.1% versus 30.2%).

**Table 1:** Pre-weighting characteristics of patients undergoing THR

	Conventional surgery (n = 919,391)	Computer guidance (n = 4,006)	Standardised mean difference
Number of revisions	19,799	72	–
Median Observation Time, years (revision or censoring) (IQR)	6.07	4.73	–
Mean age, years (SD)	69.8 (10.3)	67.6 (10.9)	−0.24
*Sex*			
Female	60.6%	56.2%	0.10
Male	39.4%	43.8%	
*ASA classification*			
1	13.7%	19.6%	−0.21
2	70.1%	70.3%	
3	15.7%	10.0%	
4	0.4%	0.2%	
5	0.0%	0.0%	
*Position*			
Lateral	93.4%	95.9%	–0.15
Supine	6.6%	4.1%	
*Approach*			
Anterior	0.1%	2.6%	0.56
Antero-lateral	1.9%	1.7%	
Direct Anterior	0.2%	0.3%	
Hardinge	22.3%	5.8%	
Hardinge/Anterolateral	4.1%	1.0%	
Lateral	3.9%	8.8%	
Other	3.7%	1.7%	
Posterior	63.6%	78.0%	
Trochanteric Osteotomy	0.3%	0.1%	
*Fixation*			
Cemented	35.6%	13.4%	0.40
Cementless	39.3%	59.4%	
Hybrid	22.2%	26.5%	
Reverse Hybrid	2.8%	0.7%	
*Bearing*			
Ceramic on Ceramic	14.0%	9.1%	−0.07
Ceramic on Metal	0.2%	0.3%	
Ceramic on Polyethylene	23.8%	38.0%	
Metal on Ceramic	0.0%	0.0%	
Metal on Polyethylene	62.0%	52.7%	
*Operation funding and hospital setting*			
Public/Public	60.4%	44.0%	0.71
Public/Private	24.8%	15.9%	
Private/Public	1.2%	0.9%	
Private/Private	13.5%	39.1%	
*BMI*			
Mean (SD)	28.7 (5.2)	28.2 (4.9)	−0.06
Availability (%)	69.9%	69.8%	
Mean Surgeon Operations per year (SD)	73.0 (45.1)	115.0 (62.4)	0.94

Post-weighting, the comparison groups were balanced in covariate distributions as illustrated in Table 2.

**Table 2:** Post-weighting characteristics of the patients within the comparison groups

	Conventional surgery versus computer guidance	Standardised mean difference
Effective Sample Size	7,235	
Mean age, years (SD)	69.4 vs 69.4 (10.2 vs 10.2)	−0.01
*Sex*		
Female	59.6% vs 59.6%	−0.01
Male	40.4% vs 40.4%	
*ASA classification*		
1	16.5% vs 16.5%	0.01
2	69.6% vs 69.6%	
3	13.6% vs 13.6%	
4	0.3% vs 0.3%	
5	0.0% vs 0.0%	
*Position*		
Lateral	94.1% vs 93.2%	0.04
Supine	5.9% vs 6.8%	
*Approach*		
Anterior	0.1% vs 0.1%	0.02
Antero-lateral	3.5% vs 3.5%	
Direct Anterior	0.5% vs 0.5%	
Hardinge	8.8% vs 8.8%	
Hardinge/Anterolateral	1.8% vs 1.8%	
Lateral	8.4% vs 8.4%	
Other	3.0% vs 3.0%	
Posterior	73.7% vs 73.7%	
Trochanteric Osteotomy	0.2% vs 0.2%	
*Fixation*		
Cemented	18.7% vs 18.7%	0.04
Cementless	58.9% vs 58.9%	
Hybrid	21.7% vs 21.7%	
Reverse Hybrid	0.8% vs 0.8%	
*Bearing*		
Ceramic on Ceramic	13.6% vs 13.6%	−0.03
Ceramic on Metal	0.2% vs 0.2%	
Ceramic on Polyethylene	30.4% vs 30.3%	
Metal on Ceramic	0.0% vs 0.0%	
Metal on Polyethylene	55.8% vs 55.9%	
*Operation funding and hospital setting*		
Public/Public	50.1% vs 50.1%	−0.01
Public/Private	25.1% vs 25.1%	
Private/Public	1.1% vs 1.1%	
Private/Private	23.7% vs 23.7%	
*BMI*		
Mean (SD)	28.9 vs 28.2 (10.6 vs 5.4)	−0.08
Mean Surgeon Operations per year	87.7 vs 87.7 (48.6 vs 56.2)	0.04

### Outcome measures

The co-primary analyses were revision for all-causes and dislocation following THR performed using computer guidance versus conventional technique.

Secondary analyses included revision for all-causes in patients aged over and under 60 years, for prosthetic joint infection, and for causes other than dislocation and prosthetic joint infection.

Additional secondary analyses were differences in patient reported joint function and health-related quality of life measured using OHS and EQ-5D-3L respectively at six months post-operatively.

Approximately 30% of THR records within the NJR dataset lack body mass index (BMI) data, the majority of these occur in the early years of the NJR. Given this potential confounder, we explored the effects of missing data through a sensitivity analysis that also considered this covariate for the comparisons revision for all-causes and PROMs [27–30]. We excluded patients from these analyses whose BMI values were outside the range of 15 to 65, considering such values erroneous. Further sensitivity analyses for revision for all-causes were conducted exploring the effects of femoral head size (< 32 mm, 32 mm, > 32 mm) and variations in implant performance profiles by restricting to the five most commonly used combination of prosthesis brands, both factors have been shown to influence revision risk [31, 32].

The occurrence of intra-operative complications between groups was also investigated.

### Statistical analysis

Propensity scores were estimated using a logistic regression model approach with Sturmer weight trimming to improve the accuracy and precision of estimates. For revision outcomes, the covariates were age, sex, American Society of Anaesthesiologists (ASA) classification, operation funding, year of surgery, approach, hospital setting (public or private), bearing, fixation, and surgeon case volume (defined as the mean number of procedures per year; analysed as a continuous measure and capped at 200 procedures/year) [33]. For PROMs outcomes, the latter three variables were substituted for pre-operative EQ-5D-3L and OHS scores as they have not been shown to influence these outcomes [34–36]. Propensity score-based weights were generated for the patient groups. Standardized mean differences were examined prior to and following the construction of weights to assess for covariate imbalance between groups. These are computed by dividing the difference in the means of the variable in the two groups by an estimate of the standard deviation. Larger values indicate that the two groups are dissimilar, a commonly recommended threshold value is < 0.1 [37]. Revision outcomes were analysed using Kaplan–Meier survival analysis to account for censoring due to death or absence of experiencing the revision event. Cases were censored by date of last follow-up or death as pre-matched to ONS data by NJR, with additional deaths identified through subsequent matching to ONS data, whichever occurred earliest. A Cox proportional hazards models was used to assess for differences in revision risk. Proportionality was explored using flexible parametric modelling to decide the most appropriate approach and comparisons were performed using likelihood ratio testing [38]. The data was modelled using restricted cubic splines with three knots to explore the possibility of a time varying effect of computer guided surgery. This model was compared to the equivalent model with no time-varying effect and found no significant difference (*p* = 0.859). Hence, Cox proportional hazards models were used, with fixed effects for surgical technique (computer guided or conventional surgery), sex, age, year of surgery, and surgeon case volume and stratified for ASA classification, approach, bearing, fixation, operation funding and hospital setting to account for potential non-proportional hazards in these groups. For the PROMs analyses, the NHS Digital case mix adjustment methodology (version three) was used to estimate the expected post-operative scores [22]. This accounts for several additional confounders amongst the population such as ethnicity. The difference between the expected and observed PROMs change scores between patient groups were analysed using a generalised linear model. The same statistical approaches were applied in the sensitivity analyses. Due to few events, an unadjusted analysis of intra-operative complications was performed using the Chi-squared test. Revision and mortality outcomes were expressed using hazard ratios (HR) while PROMs were expressed using their respective units. Effective sample sizes (ESS) are provided, reporting a comparable level of statistical power to an unweighted sample [39]. 95% confidence intervals (CI) are presented and statistical significance was set at *p* < 0.05. Analyses were carried out using Stata (version 16.1, StataCorp LP, College Station, Texas, USA, 1985–2019).

## Results

### Revision for all-causes, dislocation, and other indications

**Fig. 2:**
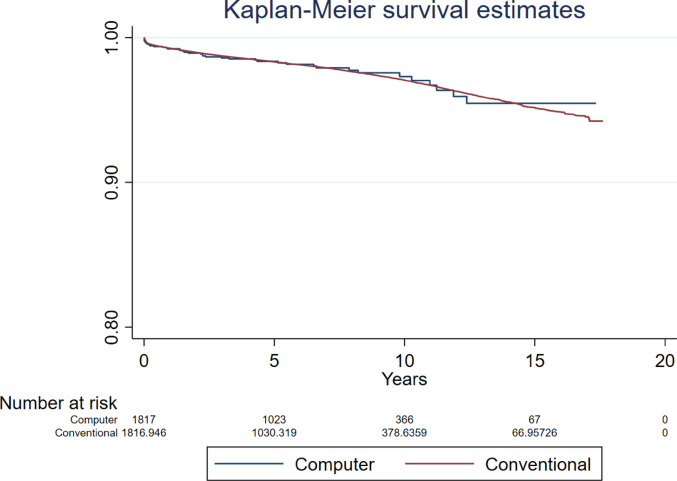
Revision for all-causes following primary THR performed using computer guidance versus conventional technique

**Fig. 3:**
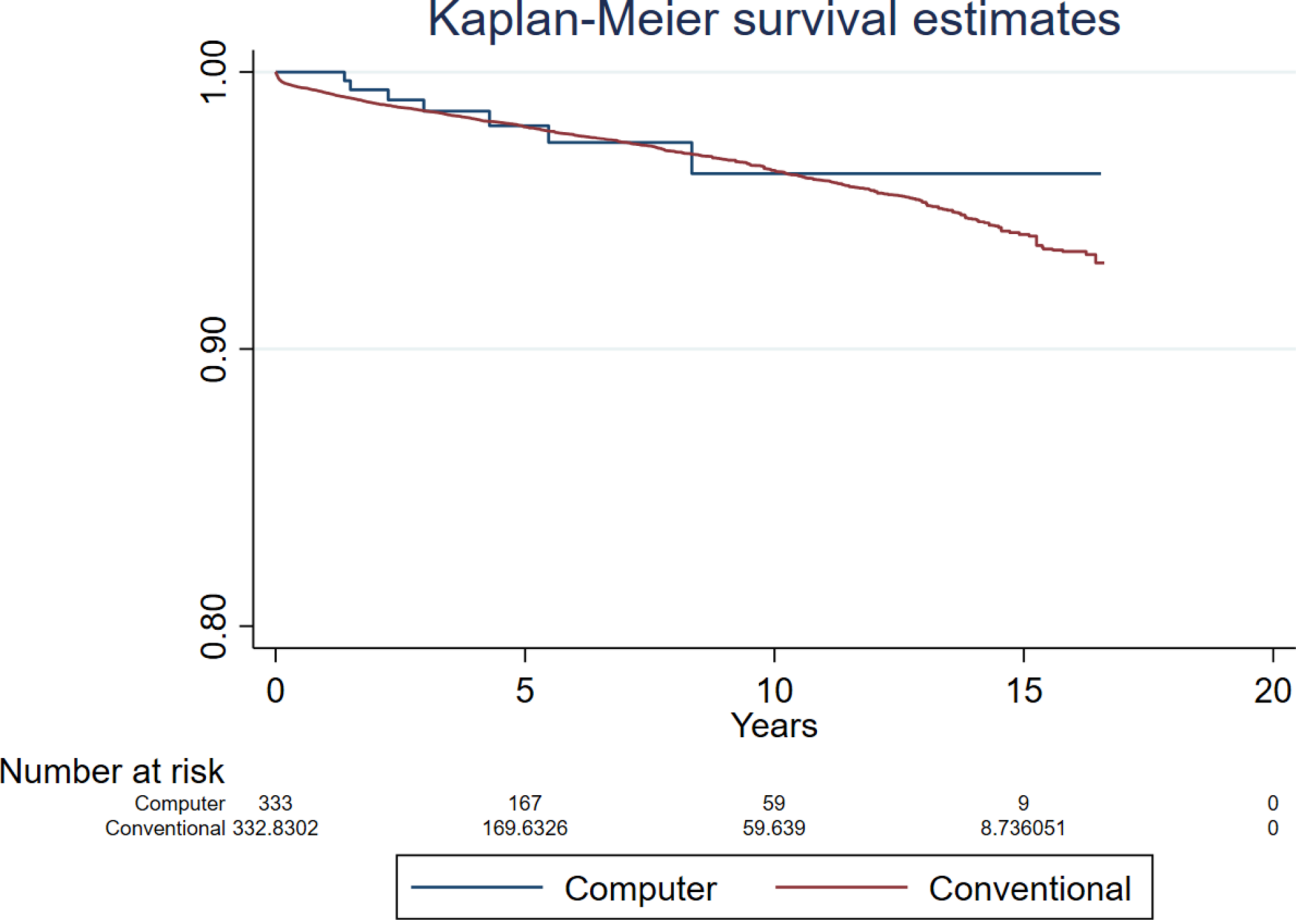
Revision for all-causes following primary THR performed using computer guidance versus conventional technique in patients younger than 60 years

**Fig. 4:**
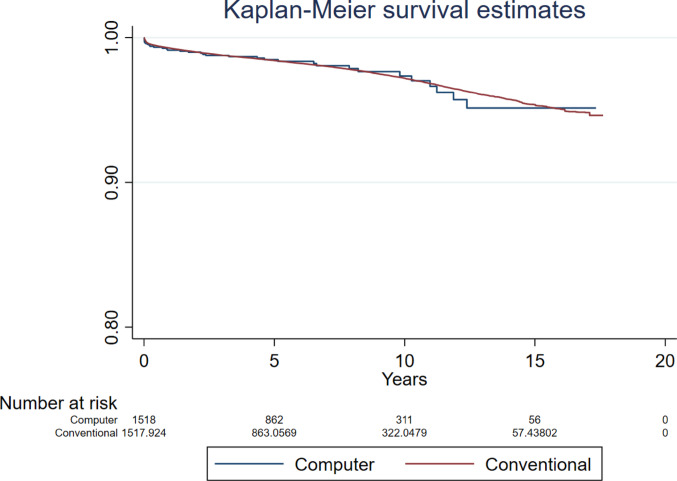
Revision for all-causes following primary THR performed using computer guidance versus conventional technique in patients older than 60 years

Compared to conventional surgery, the hazard ratio (HR) for revision for all-causes following computer guided THR was 0.947 (95% CI 0.698 to 1.283, *p* = 0.726, ESS 7,235) (Fig. 2). The analyses investigating revision for all-causes in patients aged below and over 60 years found no difference between groups (HR 0.543, 95% CI 0.220 to 1.339, *p* = 0.185, ESS 1,324, and HR 0.839, 95% CI 0.514 to 1.369, *p* = 0.482, ESS 6,071, respectively) (Figs. 3 and 4).

**Fig. 5:**
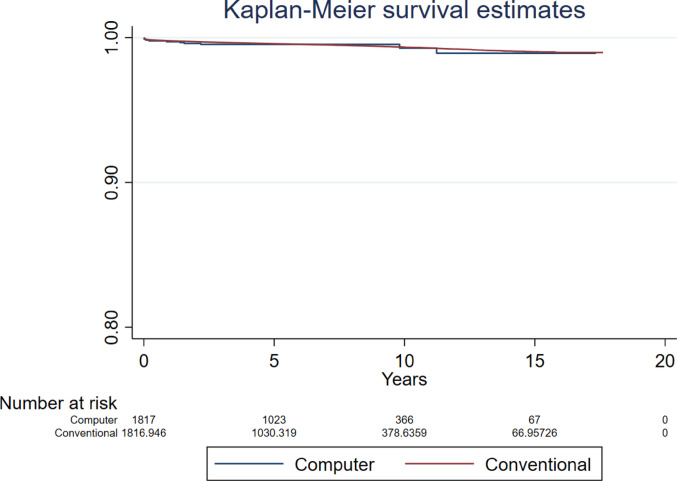
Revision for dislocation following primary THR performed using computer guidance versus conventional technique

**Fig. 6:**
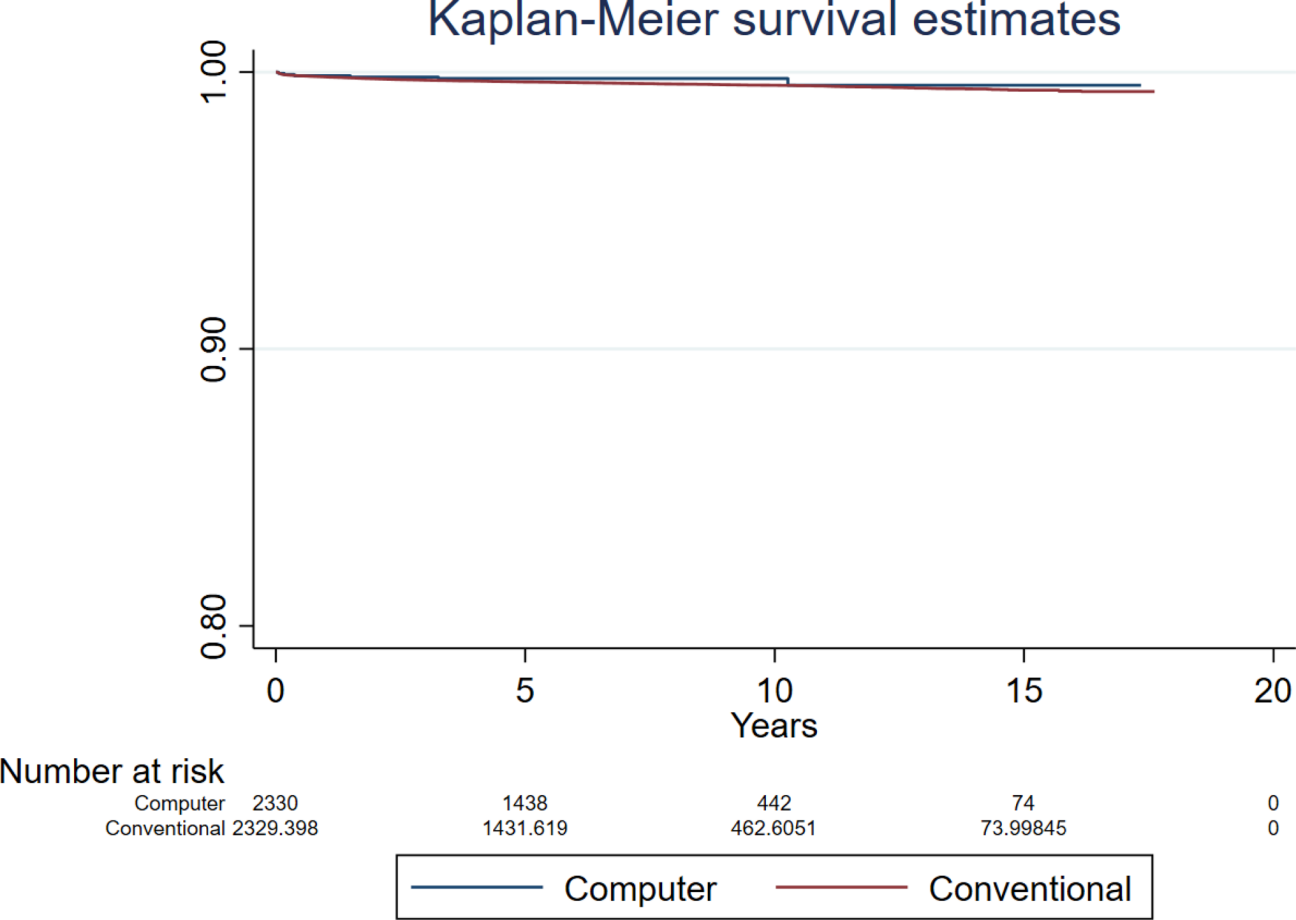
Revision for prosthetic joint infection following primary THR performed using computer guidance versus conventional technique

**Fig. 7:**
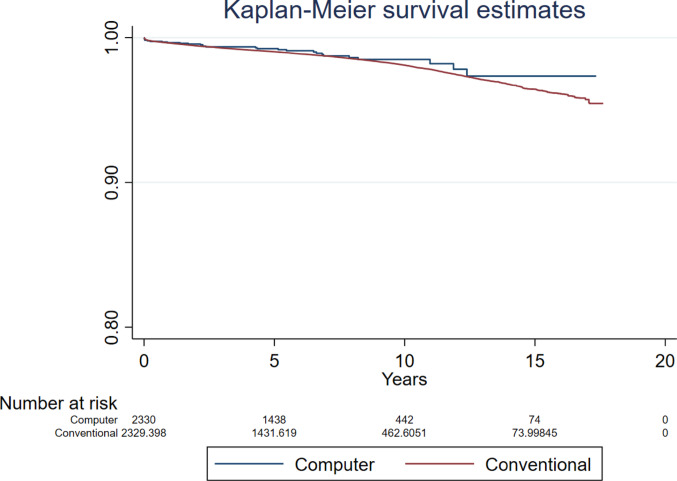
Revision for indications other than dislocation and prosthetic joint infection following primary THR performed using computer guidance versus conventional technique

There were also no differences between computer guided and conventional THR for analyses investigating revision for dislocation (HR 0.929, 95% CI 0.512 to 1.688, *p* = 0.810, ESS 7,235) (Fig. 5), prosthetic joint infection (HR 0.693, 95% CI 0.304 to 1.580, *p* = 0.384, ESS 7,235) (Fig. 6), and indications other than dislocation and prosthetic joint infection (HR 1.00, 95% CI 0.663 to 1.524, *p* = 0.982, ESS 7,235) (Fig. 7).

**Fig. 8:**
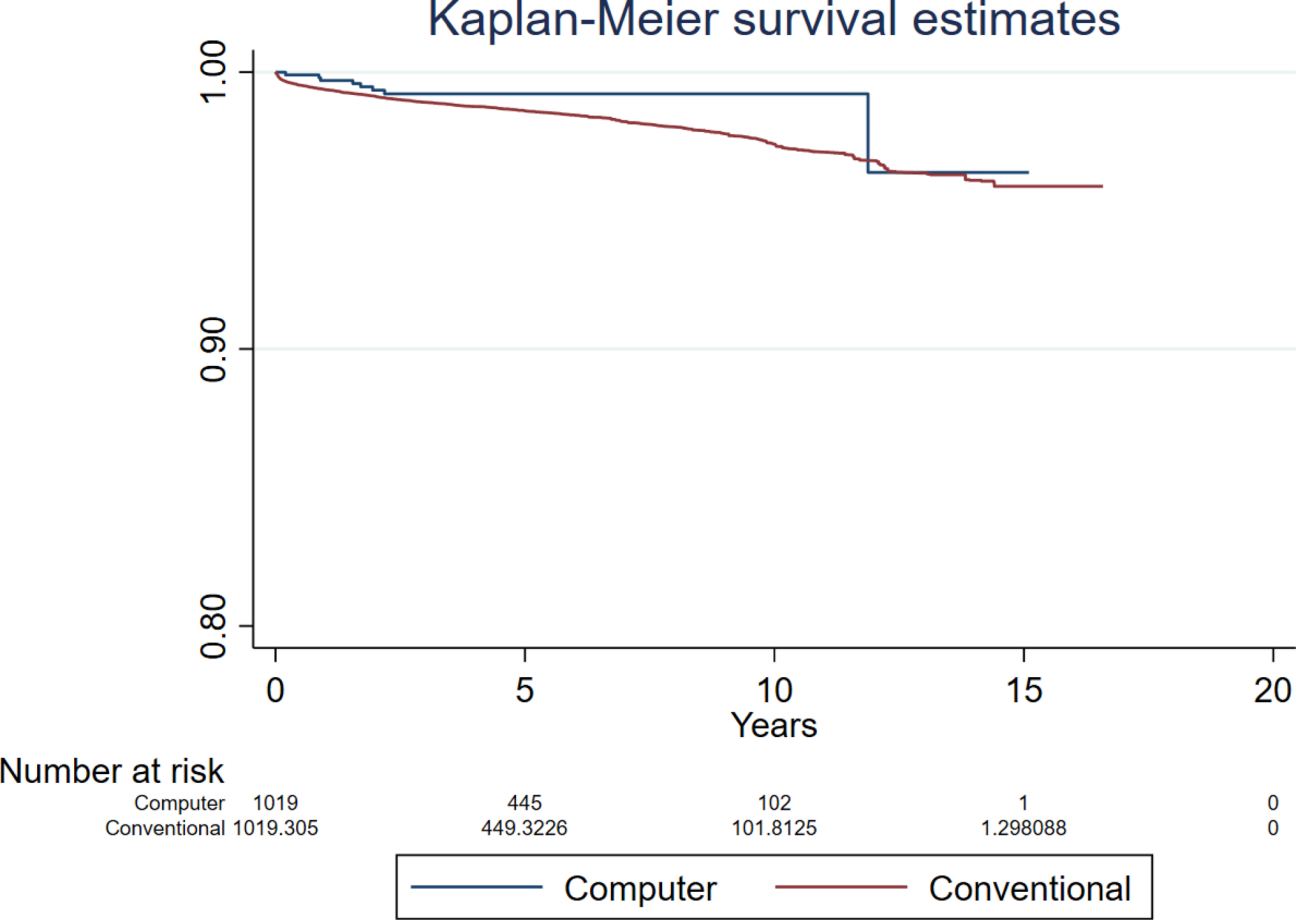
Revision for all-causes following primary THR performed using computer guidance versus conventional technique when restricting to the five most commonly used combination of prosthesis brands

The sensitivity analyses for revision for all-causes which accounted for covariates BMI and femoral head size in the model found no differences between groups (HR 0.847, 95% CI 0.564 to 1.266, *p* = 0.413, ESS 4,822, and HR 0.748, 95% CI 0.488 to 1.148, *p* = 0.184, ESS 6909, respectively) (Supplementary Figs. 1 and 2). However, there was a relatively reduced risk of revision in favour of computer guidance in the sensitivity analysis restricting to the five most commonly used combination of prosthesis brands (HR 0.446, 95% 0.231 to 0.858, *p* = 0.016, ESS 3993) (Fig. 8). This analysis included the following combination of acetabular and femoral prostheses brands: Trident and Exeter V40 (Stryker), R3 Cementless and Synergy Cementless (Smith + Nephew), Pinnacle and Corail (DePuy), R3 Cementless and Polarstem Cementless (Smith + Nephew), and Trident and Accolade II (Stryker).

### Health-related quality of life and Oxford Hip Score

Univariable regression analysis of the weighted and case mix adjusted groups revealed no differences in the change in EQ-5D scores following THR performed using computer guidance compared to conventional technique (Table 3). For OHS, there was a larger improvement observed following THR performed using computer guidance compared to conventional technique on univariable regression (Table 3).

**Table 3:** Pre- and post-operative OHS and EQ-5D scores, and regression analysis comparing conventional surgery to computer guidance. # weighted and case mix adjusted *indicates constant term in regression model

	Conventional surgery	Computer guidance
	Mean (SD) or Mean (95% CI; p value)	Mean (SD) or Mean (95% CI; p value)
EQ5D: n (unweighted)	302,859	935
Mean weighted pre-operative and post-operative scores (SD)	0.371 (0.317) and 0.813 (0.235)	0.371 (0.314) and 0.820 (0.235)
Univariable # [ESS]	*	+ 0.007 (−0.008 to 0.023; *p* = 0.356) [2929]
OHS: n (unweighted)	328,634	1,001
Mean weighted pre-operative and post-operative (SD)	18.570 (8.063) and 40.354 (8.340)	18.569 (7.966) and 40.945 (8.389)
Univariable # [ESS]	*	+ 0.931 (0.308 to 1.554; *p* = 0.003) [2112]

Results of the sensitivity analysis accounting for BMI in the model demonstrated similar results to the primary analyses and are shown in supplementary Table 1.

### Intra-operative complications

The incidence of intra-operative complications was significantly fewer in the computer guided THR group (0.51% versus 0.96%, *p* = 0.006) (Table 4). There was missing data for 39,056 procedures.

**Table 4:** Intra-operative complications that occurred among the patient groups

	Conventional surgery (n = 880,807)	Computer guidance (n = 3,534)
None	872,205 (99.0%)	3516 (99.5%)
Calcar Crack	2,842 (0.3%)	4 (0.1%)
Pelvic Penetration	981 (0.1%)	2 (< 0.1%)
Shaft Fracture	418 (< 0.1%)	1 (< 0.1%)
Shaft Penetration	135 (< 0.1%)	1 (< 0.1%)
Trochanteric Fracture	1,534 (0.2%)	7 (0.2%)
Other	2,692 (0.3%)	3 (< 0.1%)

## Discussion

This pragmatic study analysed several linked registry data sets and accounted for several types of confounding using propensity score-based risk adjustment statistical techniques to investigate differences in revision risk, OHS, and EQ-5D following THR performed using computer guidance compared to conventional technique. The risk of intra-operative complications was also compared between these two patient groups. There were no differences in the primary analyses for revision for all-causes and dislocation comparing these two surgical methods. However, the sensitivity analysis which restricted to the five most commonly used combination of prosthesis brands demonstrated a reduction in revision for all-causes in favour of computer guided THR. Additionally, there were relatively fewer intra-operative complications that occurred during computer guided THR. Although the sensitivity analysis evaluated a smaller sample size and shorter follow-up period, and the analysis of intra-operative complications was unadjusted, these findings suggest potential clinical benefits and indicate a possible signal of effectiveness. In terms of PROMs, there was a greater improvement in OHS favouring computer guided THR however this did not exceed the minimum clinically important difference of approximately 11 points [40, 41]. For EQ-5D there were no differences between patient groups.

Few published studies have investigated this topic using large datasets. Davis et al. [42] analysed the NJR (of England and Wales) and PROMs data sets comparing all-cause revision, OHS, and EQ-5D following computer guided and conventional THR. Their study found a reduction in all-cause revision using computer guidance (HR 0.45, 95% CI 0.21 to 0.96, *p* = 0.038) however their results for OHS (40.5, 95% CI 39.7 to 41.2, versus 39.7, 95% CI 39.6 to 39.9; *p* = 0.11) and EQ-5D (0.814, 95% CI 0.791 to 0.836, versus 0.798, 95% CI 0.793 to 0.802; *p* = 0.3) were similar between the two patient groups. In contrast to our study, eligible procedures were restricted to uncemented and hybrid fixations, and components of a single manufacturer only. Using the Australian National Joint registry data set, Agarwal et al. found a reduced risk of revision for dislocation following navigated compared to non-navigated THR (HR 0.46; 95% CI 0.29 to 0.74, *p* = 0.002). However, no difference in revision for all-causes was observed between groups at the same timepoint (HR 0.89; 95% CI 0.76 to 1.04, *p* = 0.138) [20]. Similar to our study, the authors also conducted an analysis including only the five most commonly used acetabular and femoral components which found a reduced risk of all-cause revision (HR 0.64; 95% CI 0.48 to 0.86, *p* = 0.003). Differences in results for revision for dislocation may be attributed to characteristic differences between patient groups relating to this complication’s multifactorial aetiology [43–46].

Strengths of our study include the use of a national registry data set which allowed one of the largest analyses of computer navigated THR procedures to be performed. Furthermore, patients were followed up over a long period of time and procedures were performed by a large group of surgeons across many centres meaning our results are reflective of general practice. The use of propensity scores allowed comparable patient groups to be generated, accounting for their probability of receiving either of the interventions based on a range of variables [47]. This helped improve the study quality by limiting confounding by indication however this approach also affected the effective sample sizes for the analyses. For this reason and the limited use of computer guidance over the study period, our analyses comparing revision events were underpowered. A sample size calculation performed determined that to detect a hazard ratio of 0.9 in all-cause revision with alpha set at 0.05 and 80% power, assuming 1:1 allocation of participants, would need 62,850 patients in total to be followed up for 20 years post-operatively. This large figure and long follow-up period needed most likely precludes a randomised controlled trial investigating this outcome, and our study design is therefore the most feasible to answer this research question by utilising a large data set due to revision being a rare outcome [48]. In addition to revision outcomes, we also compared PROMs including OHS and EQ-5D which is another major strength to our study. The use of NHS Digital case mix adjustment helped improve the validity of the findings by accounting for variation in the characteristics and comorbidities of the population, and which can affect PROMs such as deprivation and depression respectively [22, 49].

It is important to mention the limitations inherent to an observational study design within the context of our research. Despite application of a variety of statistical techniques, there remained potential for confounding by indication given it was not possible to adjust for unmeasured variables not captured within the NJR dataset such as case complexity or other factors which may have necessitated the surgeon to use computer guidance during the procedure. Moreover, residual confounding cannot be excluded, as additional factors including spinopelvic alignment, patients’ postoperative expectations and functional demands, and variations in surgical technique may also have influenced the observed results. There are also a variety of computer guidance systems available for use and differential performance between these systems is a possibility. Certain systems are image based requiring cross sectional imaging prior to the operation and this information itself may have provided additional support to the surgeon performing the case [50]. Furthermore, certain systems constrain the surgeon to implant specific prosthesis and which do not necessarily have established performance profiles [51, 52]. For this reason, we conducted a sensitivity analysis that explored the effects of revision for all-causes restricting to the five most commonly used prosthesis brands however this affected the sample size. There are also some limitations associated with PROMs analyses such as the reduced generalisability of its findings due to approximately 60% of procedures overall having missing data [4]. Although there was a slightly greater proportion of missing PROMs data in the computer guided patient group, this is unlikely to be due to dissatisfaction with the outcome of their THR [53].

## Conclusions

Our study did not find definitive evidence of a reduction in revision risk or clinically meaningful improvements in patient reported outcomes following THR using computer navigation technology compared to conventional technique. However, a reduced risk of intraoperative complications was observed in the computer guided group, although this finding is based on an unadjusted analysis. In a sensitivity analysis limited to the five most commonly used combination of prosthesis brands, we found a reduction in revision risk in favour of computer guided THR. However, this finding pertains to a specific subset of patients and is limited by a smaller sample size and shorter follow up duration, which precludes definitive conclusions to support a change in clinical practice. Nonetheless, both this finding and that of intra-operative complications indicate early signals of effectiveness, which support the need for further research using larger cohorts and extended follow up. The modest improvement in OHS with the use of computer navigation was not clinically meaningful, and there were no differences found for EQ-5D. It is important to interpret our findings considering the study’s limitations, including those inherent to an observational design, particularly the potential for confounding, as well as the constraints associated with analyses of large registry datasets. Lastly, it is important to mention that the evaluation of a new technology presents challenges when its content and methods are not static but continue to evolve. Retrospective cohort analyses such as this study inevitably evaluate the past rather than the present. While an effective summary of currently available evidence is important, the authors acknowledge it is unlikely that computer guided surgery technologies have reached final maturity.

## Supplementary Information

Below is the link to the electronic supplementary material.

Supplementary Material 1

Supplementary Material 2

Supplementary Material 3
