# Supplementary material for: The effect of computer guided total hip replacement on risk of revision, Oxford Hip Score, and health related quality of life: an analysis of National Joint Registry data
Source: Eur J Orthop Surg Traumatol. 2025 Dec 24;36(1):51. doi: 10.1007/s00590-025-04622-9 (PMC12738641; doi:10.1007/s00590-025-04622-9)

Supplementary figure 1: Revision for all-causes following primary THR performed using computer guidance versus conventional technique when also accounting for BMI in the model


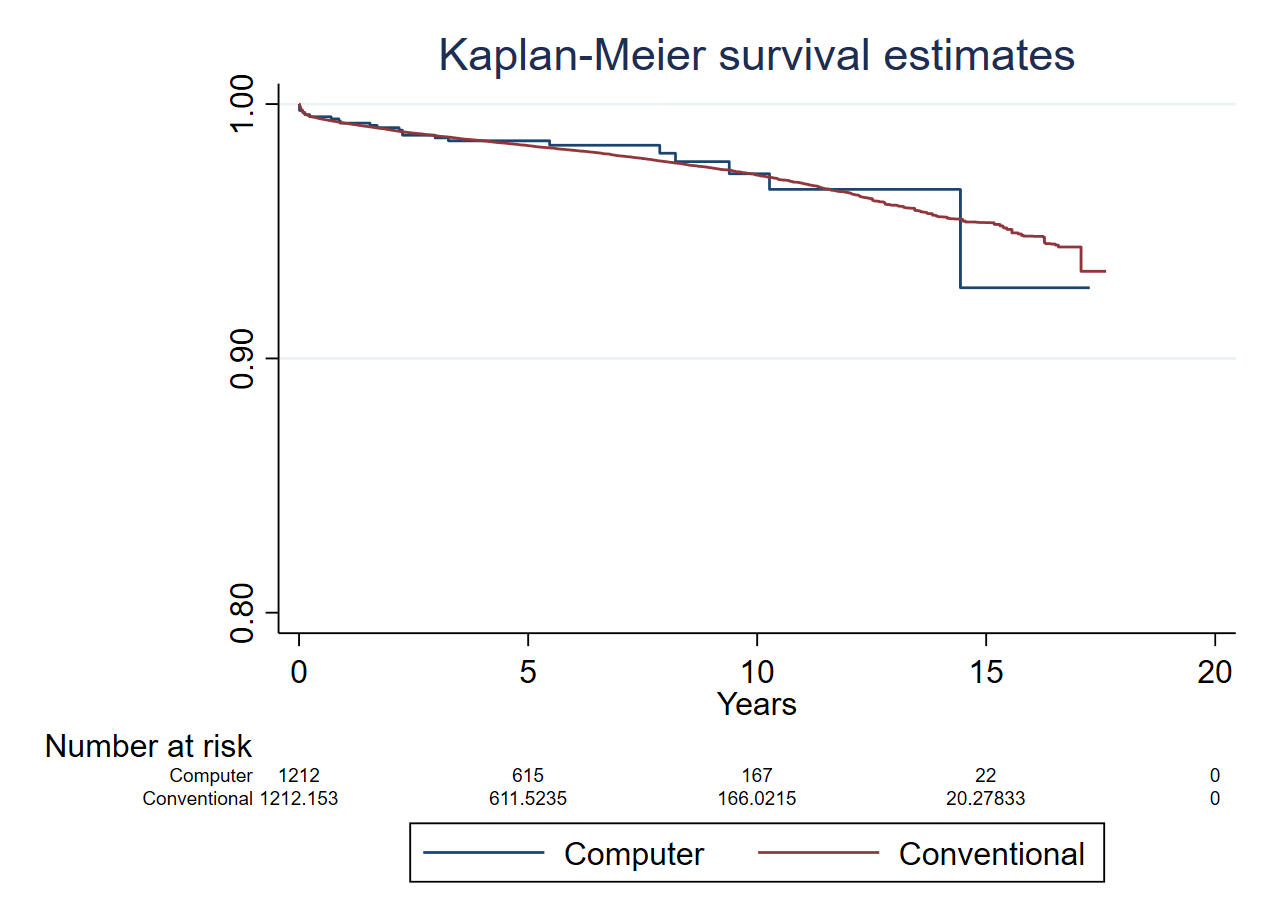

Supplement: Supplementary file 1 — Supplementary Material 1 [file 590_2025_4622_MOESM1_ESM.docx]
