# Supplementary material for: The effect of computer guided total hip replacement on risk of revision, Oxford Hip Score, and health related quality of life: an analysis of National Joint Registry data
Source: Eur J Orthop Surg Traumatol. 2025 Dec 24;36(1):51. doi: 10.1007/s00590-025-04622-9 (PMC12738641; doi:10.1007/s00590-025-04622-9)
Supplement: Supplementary file 3 — Supplementary Material 3 [file 590_2025_4622_MOESM3_ESM.docx]

|  | **Conventional surgery** | **Computer guidance** |
| --- | --- | --- |
|  | **Mean (SD) or Mean (95% CI; p value)** | **Mean (SD) or Mean (95% CI; p value)** |
| **EQ-5D-3L: n**  **(unweighted)** | 234,738 | 716 |
| **Pre-operative (weighted)** | 0.369 (0.317) | 0.369 (0.316) |
| **Post-operative (weighted)** | 0.817 (0.233) | 0.829 (0.227) |
| **Univariable (weighted and case mix adjusted) [ESS]** | * | +0.011 (-0.006 to 0.027; p=0.212)  [2342] |
| **OHS: n**  **(unweighted)** | 254,587 | 766 |
| **Pre-operative (weighted)** | 18.570 (8.055) | 18.569 (8.088) |
| **Post-operative (weighted)** | 40.542 (8.211) | 41.488 (7.861) |
| **Univariable (weighted and case mix adjusted) [ESS]** | * | +1.232 (0.578 to 1.886; p<0.001)  [1729] |

Supplementary table 1: Pre- and post-operative OHS and EQ-5D-3L scores, and regression analysis comparing computer guidance and conventional surgery patient groups when accounting for BMI in the model. *indicates constant term in regression model
